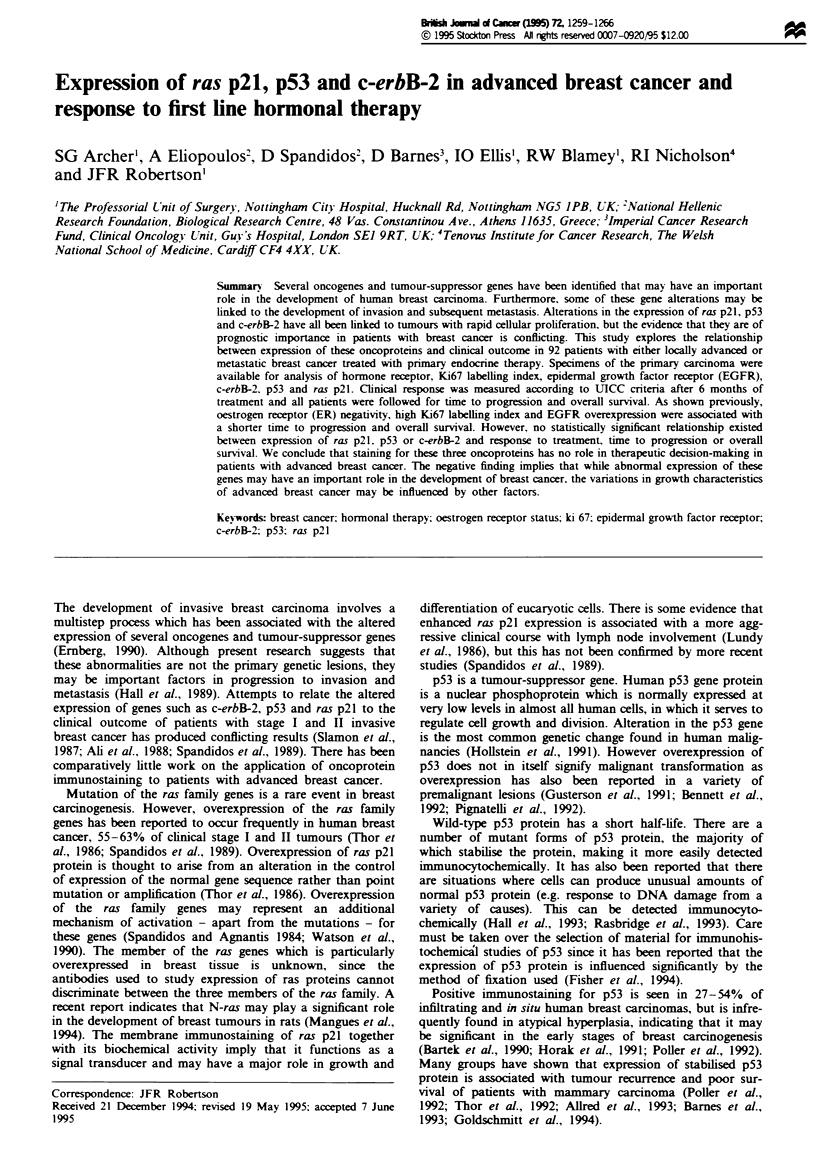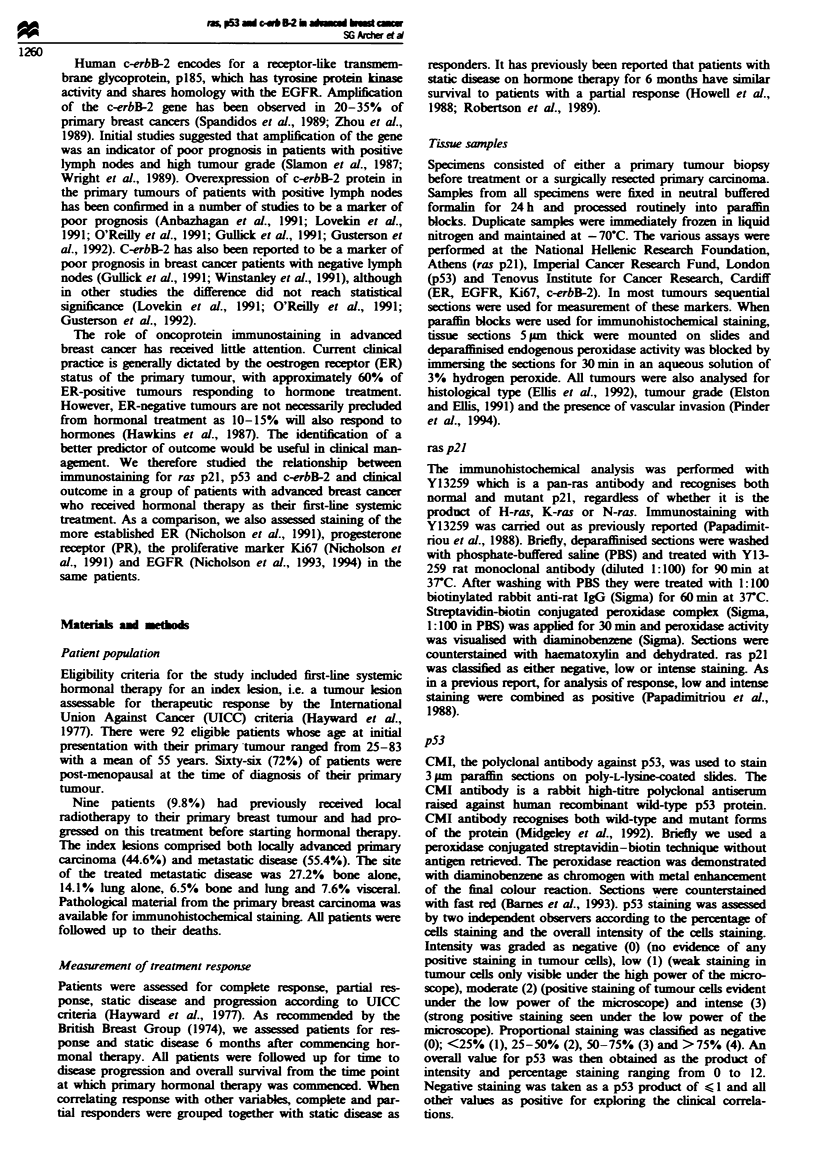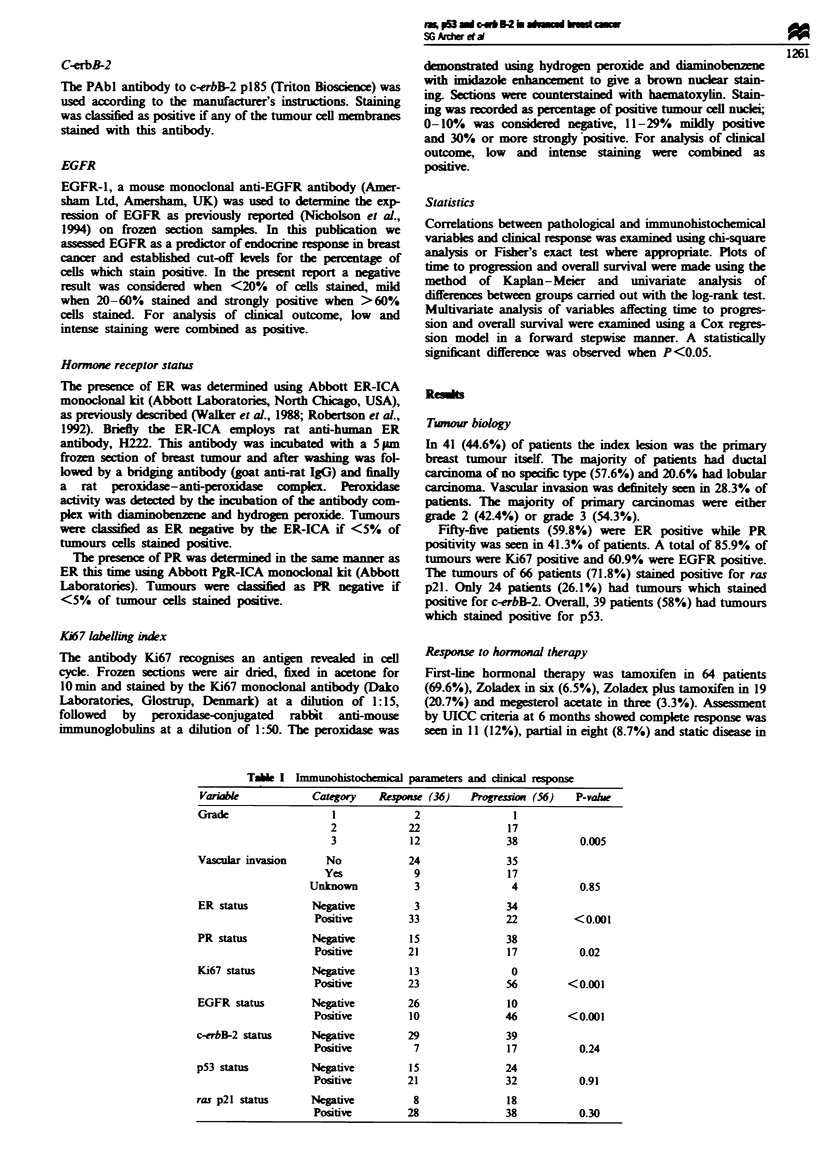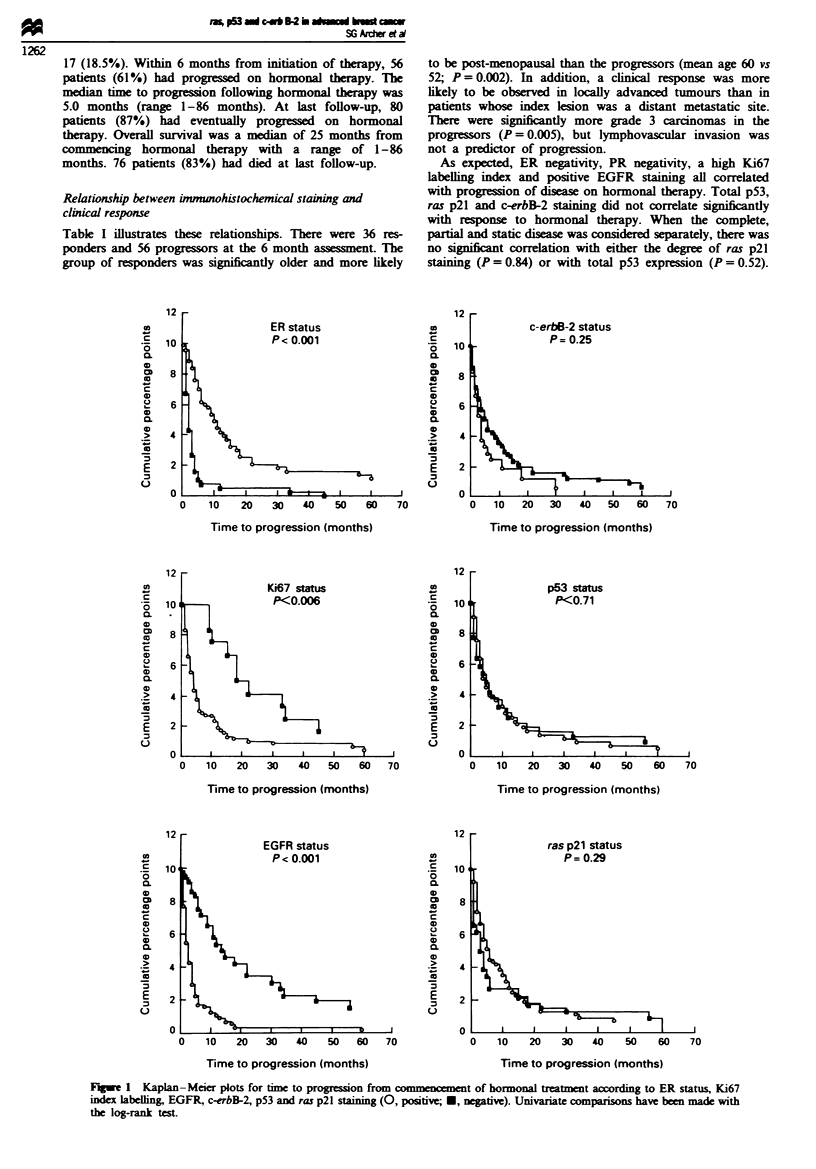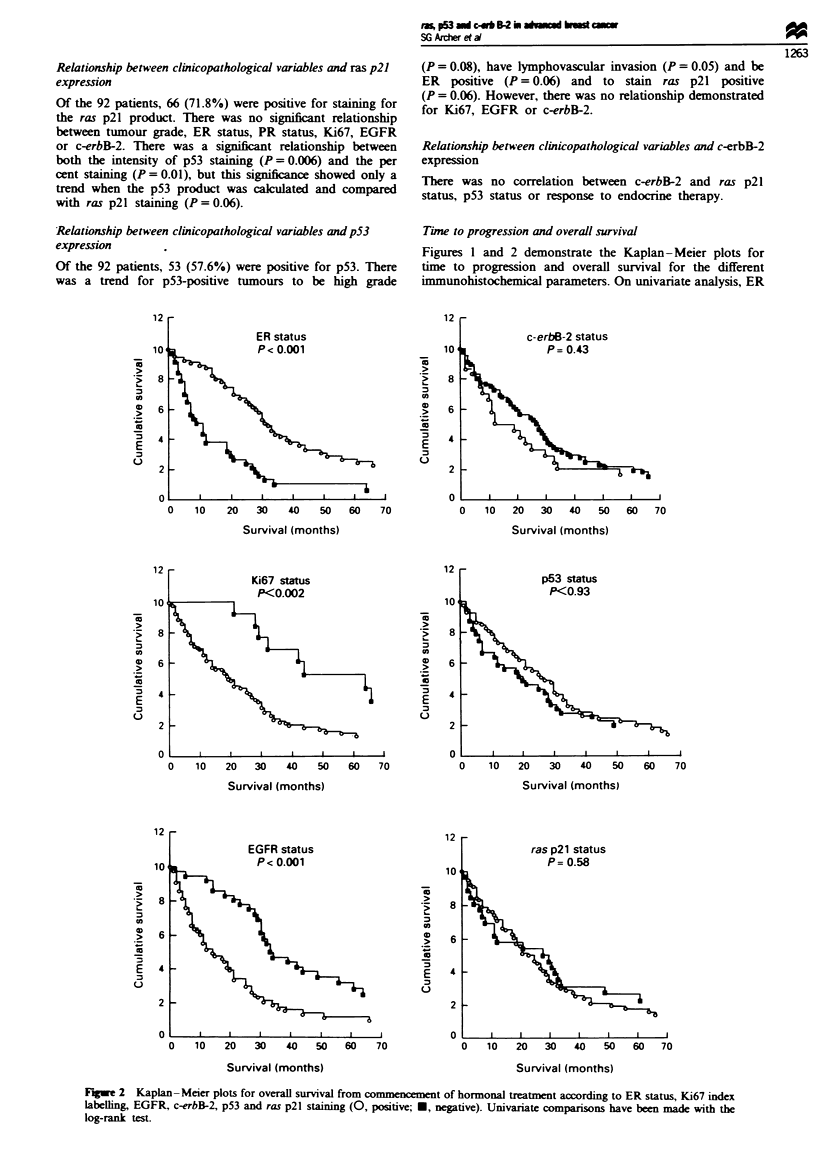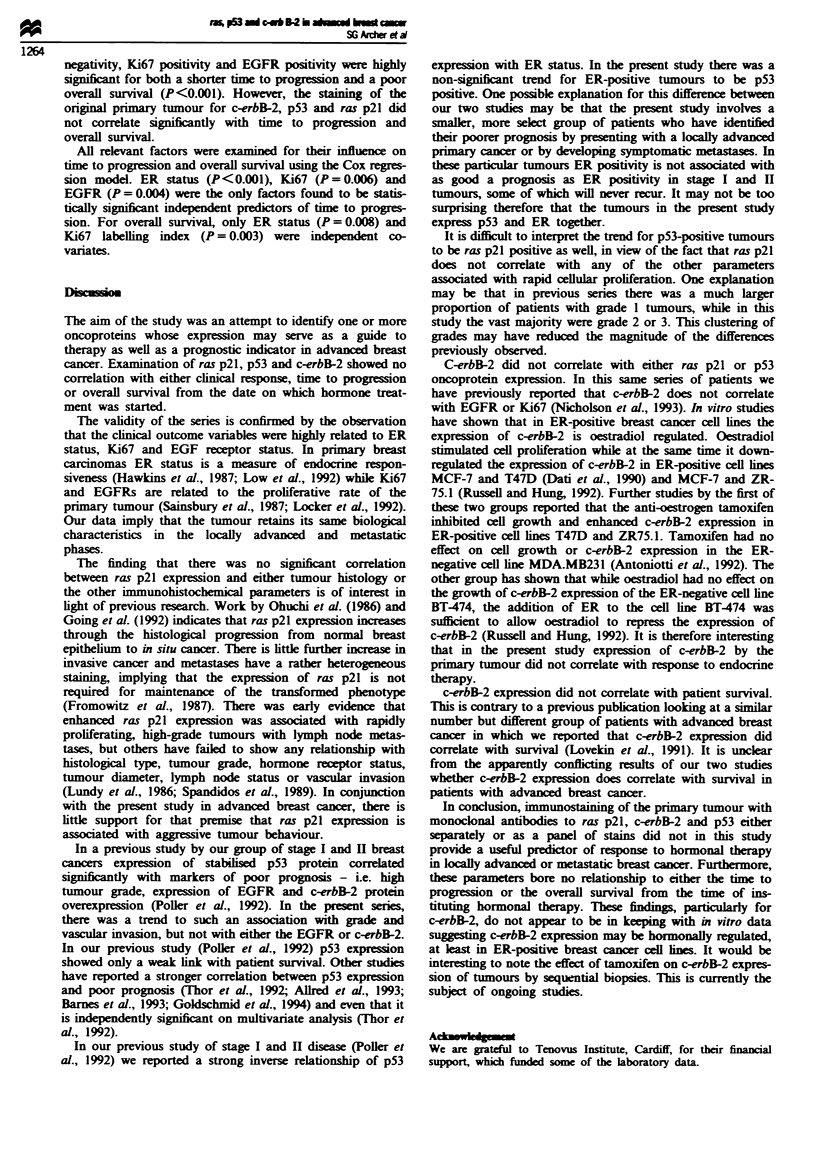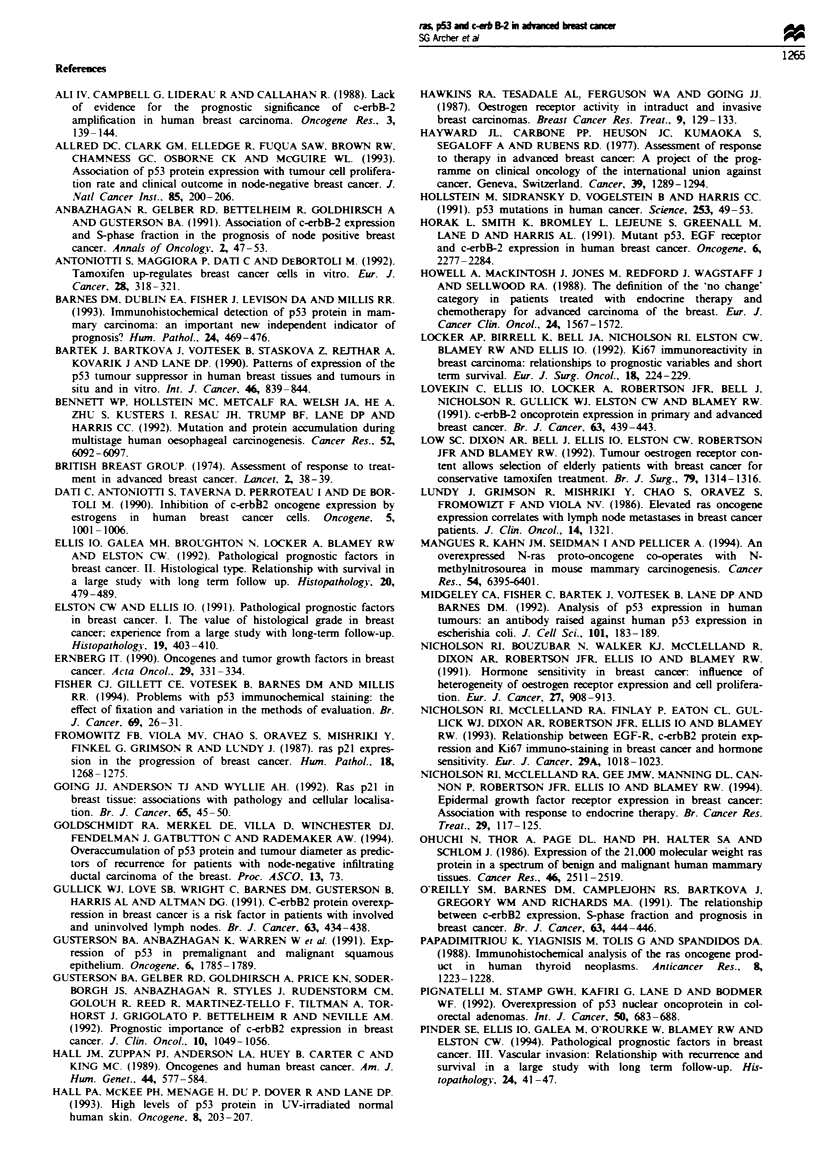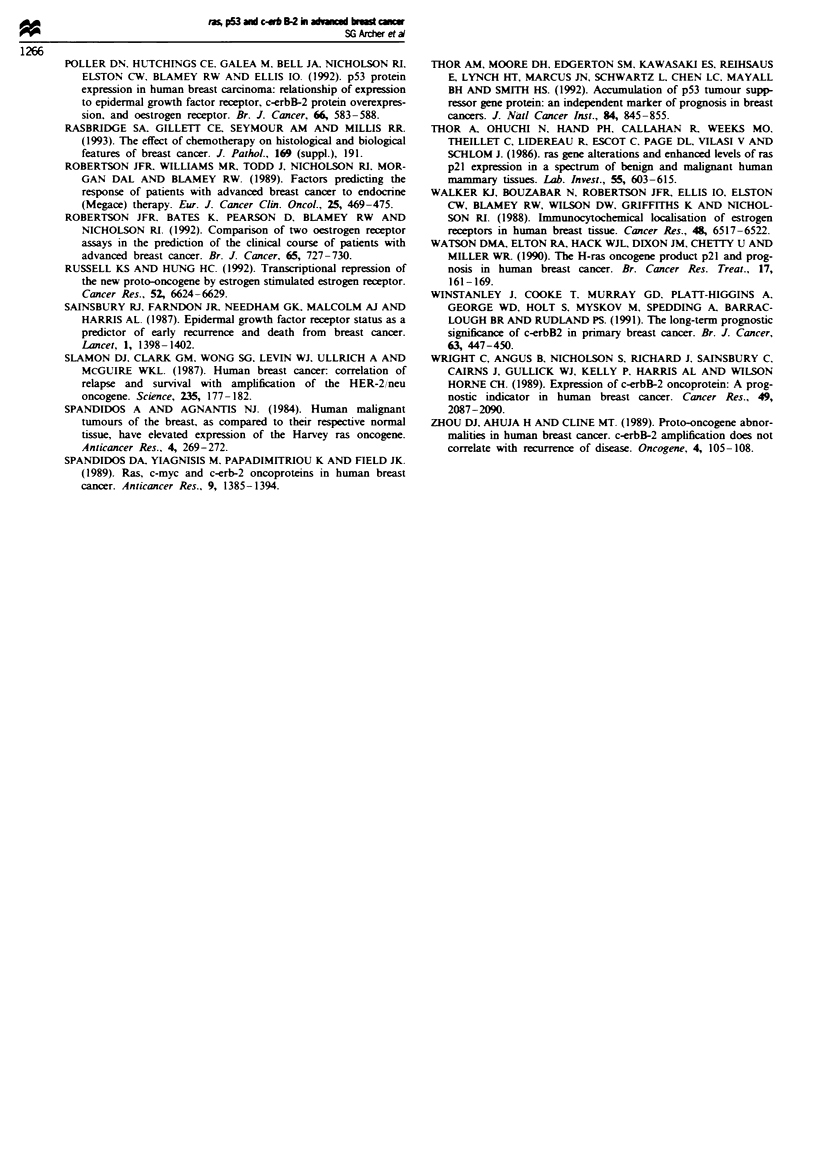# Expression of ras p21, p53 and c-erbB-2 in advanced breast cancer and response to first line hormonal therapy.

**DOI:** 10.1038/bjc.1995.497

**Published:** 1995-11

**Authors:** S. G. Archer, A. Eliopoulos, D. Spandidos, D. Barnes, I. O. Ellis, R. W. Blamey, R. I. Nicholson, J. F. Robertson

**Affiliations:** Professional Unit of Surgery, Nottingham City Hospital, UK.

## Abstract

Several oncogenes and tumour-suppressor genes have been identified that may have an important role in the development of human breast carcinoma. Furthermore, some of these gene alterations may be linked to the development of invasion and subsequent metastasis. Alterations in the expression of ras p21, p53 and c-erbB-2 have all been linked to tumours with rapid cellular proliferation, but the evidence that they are of prognostic importance in patients with breast cancer is conflicting. This study explores the relationship between expression of these oncoproteins and clinical outcome in 92 patients with either locally advanced or metastatic breast cancer treated with primary endocrine therapy. Specimens of the primary carcinoma were available for analysis of hormone receptor, Ki67 labelling index, epidermal growth factor receptor (EGFR), c-erbB-2, p53 and ras p21. Clinical response was measured according to UICC criteria after 6 months of treatment and all patients were followed for time to progression and overall survival. As shown previously, oestrogen receptor (ER) negativity, high Ki67 labelling index and EGFR overexpression were associated with a shorter time to progression and overall survival. However, no statistically significant relationship existed between expression of ras p21, p53 or c-erbB-2 and response to treatment, time to progression or overall survival. We conclude that staining for these three oncoproteins has no role in therapeutic decision-making in patients with advanced breast cancer. The negative finding implies that while abnormal expression of these genes may have an important role in the development of breast cancer, the variations in growth characteristics of advanced breast cancer may be influenced by other factors.


© 1995 Stockton Press All rights reserved 0007-0920/95 $12.00

### Expression of *ras* p21, p53 and c-*erb*B-2 in advanced breast cancer and response to first line hormonal therapy

SG Archer[1], A Eliopoulos[2], D Spandidos[2], D Barnes[3], IO Ellis[1], RW Blamey[1], RI Nicholson[4] and JFR Robertson[1]

[1]*The Professorial Unit of Surgery, Nottingham City Hospital, Hucknall Rd, Nottingham NG5 1PB, UK;* [2]*National Hellenic Research Foundation, Biological Research Centre, 48 Vas. Constantinou Ave., Athens 11635, Greece;* [3]*Imperial Cancer Research Fund, Clinical Oncology Unit, Guy's Hospital, London SE1 9RT, UK;* [4]*Tenovus Institute for Cancer Research, The Welsh National School of Medicine, Cardiff CF4 4XX, UK.*

**Summary** Several oncogenes and tumour-suppressor genes have been identified that may have an important role in the development of human breast carcinoma. Furthermore, some of these gene alterations may be linked to the development of invasion and subsequent metastasis. Alterations in the expression of *ras* p21, p53 and c-*erb*B-2 have all been linked to tumours with rapid cellular proliferation, but the evidence that they are of prognostic importance in patients with breast cancer is conflicting. This study explores the relationship between expression of these oncoproteins and clinical outcome in 92 patients with either locally advanced or metastatic breast cancer treated with primary endocrine therapy. Specimens of the primary carcinoma were available for analysis of hormone receptor, Ki67 labelling index, epidermal growth factor receptor (EGFR), c-*erb*B-2, p53 and *ras* p21. Clinical response was measured according to UICC criteria after 6 months of treatment and all patients were followed for time to progression and overall survival. As shown previously, oestrogen receptor (ER) negativity, high Ki67 labelling index and EGFR overexpression were associated with a shorter time to progression and overall survival. However, no statistically significant relationship existed between expression of *ras* p21, p53 or c-*erb*B-2 and response to treatment, time to progression or overall survival. We conclude that staining for these three oncoproteins has no role in therapeutic decision-making in patients with advanced breast cancer. The negative finding implies that while abnormal expression of these genes may have an important role in the development of breast cancer, the variations in growth characteristics of advanced breast cancer may be influenced by other factors.

**Keywords:** breast cancer; hormonal therapy; oestrogen receptor status; ki 67; epidermal growth factor receptor; c-*erb*B-2; p53; *ras* p21

The development of invasive breast carcinoma involves a multistep process which has been associated with the altered expression of several oncogenes and tumour-suppressor genes (Ernberg, 1990). Although present research suggests that these abnormalities are not the primary genetic lesions, they may be important factors in progression to invasion and metastasis (Hall *et al.*, 1989). Attempts to relate the altered expression of genes such as c-*erb*B-2, p53 and *ras* p21 to the clinical outcome of patients with stage I and II invasive breast cancer has produced conflicting results (Slamon *et al.*, 1987; Ali *et al.*, 1988; Spandidos *et al.*, 1989). There has been comparatively little work on the application of oncoprotein immunostaining to patients with advanced breast cancer.

Mutation of the *ras* family genes is a rare event in breast carcinogenesis. However, overexpression of the *ras* family genes has been reported to occur frequently in human breast cancer, 55-63% of clinical stage I and II tumours (Thor *et al.*, 1986; Spandidos *et al.*, 1989). Overexpression of *ras* p21 protein is thought to arise from an alteration in the control of expression of the normal gene sequence rather than point mutation or amplification (Thor *et al.*, 1986). Overexpression of the *ras* family genes may represent an additional mechanism of activation - apart from the mutations - for these genes (Spandidos and Agnantis 1984; Watson *et al.*, 1990). The member of the *ras* genes which is particularly overexpressed in breast tissue is unknown, since the antibodies used to study expression of ras proteins cannot discriminate between the three members of the *ras* family. A recent report indicates that N-*ras* may play a significant role in the development of breast tumours in rats (Mangues *et al.*, 1994). The membrane immunostaining of *ras* p21 together with its biochemical activity imply that it functions as a signal transducer and may have a major role in growth and differentiation of eucaryotic cells. There is some evidence that enhanced *ras* p21 expression is associated with a more aggressive clinical course with lymph node involvement (Lundy *et al.*, 1986), but this has not been confirmed by more recent studies (Spandidos *et al.*, 1989).

Correspondence: JFR Robertson
Received 21 December 1994; revised 19 May 1995; accepted 7 June 1995

p53 is a tumour-suppressor gene. Human p53 gene protein is a nuclear phosphoprotein which is normally expressed at very low levels in almost all human cells, in which it serves to regulate cell growth and division. Alteration in the p53 gene is the most common genetic change found in human malignancies (Hollstein *et al.*, 1991). However overexpression of p53 does not in itself signify malignant transformation as overexpression has also been reported in a variety of premalignant lesions (Gusterson *et al.*, 1991; Bennett *et al.*, 1992; Pignatelli *et al.*, 1992).

Wild-type p53 protein has a short half-life. There are a number of mutant forms of p53 protein, the majority of which stabilise the protein, making it more easily detected immunocytochemically. It has also been reported that there are situations where cells can produce unusual amounts of normal p53 protein (e.g. response to DNA damage from a variety of causes). This can be detected immunocytochemically (Hall *et al.*, 1993; Rasbridge *et al.*, 1993). Care must be taken over the selection of material for immunohistochemical studies of p53 since it has been reported that the expression of p53 protein is influenced significantly by the method of fixation used (Fisher *et al.*, 1994).

Positive immunostaining for p53 is seen in 27-54% of infiltrating and *in situ* human breast carcinomas, but is infrequently found in atypical hyperplasia, indicating that it may be significant in the early stages of breast carcinogenesis (Bartek *et al.*, 1990; Horak *et al.*, 1991; Poller *et al.*, 1992). Many groups have shown that expression of stabilised p53 protein is associated with tumour recurrence and poor survival of patients with mammary carcinoma (Poller *et al.*, 1992; Thor *et al.*, 1992; Allred *et al.*, 1993; Barnes *et al.*, 1993; Goldschmitt *et al.*, 1994).

Human c-*erb*B-2 encodes for a receptor-like transmembrane glycoprotein, p185, which has tyrosine protein kinase activity and shares homology with the EGFR. Amplification of the c-*erb*B-2 gene has been observed in 20–35% of primary breast cancers (Spandidos *et al*., 1989; Zhou *et al*., 1989). Initial studies suggested that amplification of the gene was an indicator of poor prognosis in patients with positive lymph nodes and high tumour grade (Slamon *et al*., 1987; Wright *et al*., 1989). Overexpression of c-*erb*B-2 protein in the primary tumours of patients with positive lymph nodes has been confirmed in a number of studies to be a marker of poor prognosis (Anbazhagan *et al*., 1991; Lovekin *et al*., 1991; O'Reilly *et al*., 1991; Gullick *et al*., 1991; Gusterson *et al*., 1992). C-*erb*B-2 has also been reported to be a marker of poor prognosis in breast cancer patients with negative lymph nodes (Gullick *et al*., 1991; Winstanley *et al*., 1991), although in other studies the difference did not reach statistical significance (Lovekin *et al*., 1991; O'Reilly *et al*., 1991; Gusterson *et al*., 1992).

The role of oncoprotein immunostaining in advanced breast cancer has received little attention. Current clinical practice is generally dictated by the oestrogen receptor (ER) status of the primary tumour, with approximately 60% of ER-positive tumours responding to hormone treatment. However, ER-negative tumours are not necessarily precluded from hormonal treatment as 10–15% will also respond to hormones (Hawkins *et al*., 1987). The identification of a better predictor of outcome would be useful in clinical management. We therefore studied the relationship between immunostaining for *ras* p21, p53 and c-*erb*B-2 and clinical outcome in a group of patients with advanced breast cancer who received hormonal therapy as their first-line systemic treatment. As a comparison, we also assessed staining of the more established ER (Nicholson *et al*., 1991), progesterone receptor (PR), the proliferative marker Ki67 (Nicholson *et al*., 1991) and EGFR (Nicholson *et al*., 1993, 1994) in the same patients.

### Materials and methods

### *Patient population*

Eligibility criteria for the study included first-line systemic hormonal therapy for an index lesion, i.e. a tumour lesion assessable for therapeutic response by the International Union Against Cancer (UICC) criteria (Hayward *et al*., 1977). There were 92 eligible patients whose age at initial presentation with their primary tumour ranged from 25–83 with a mean of 55 years. Sixty-six (72%) of patients were post-menopausal at the time of diagnosis of their primary tumour.

Nine patients (9.8%) had previously received local radiotherapy to their primary breast tumour and had progressed on this treatment before starting hormonal therapy. The index lesions comprised both locally advanced primary carcinoma (44.6%) and metastatic disease (55.4%). The site of the treated metastatic disease was 27.2% bone alone, 14.1% lung alone, 6.5% bone and lung and 7.6% visceral. Pathological material from the primary breast carcinoma was available for immunohistochemical staining. All patients were followed up to their deaths.

### *Measurement of treatment response*

Patients were assessed for complete response, partial response, static disease and progression according to UICC criteria (Hayward *et al*., 1977). As recommended by the British Breast Group (1974), we assessed patients for response and static disease 6 months after commencing hormonal therapy. All patients were followed up for time to disease progression and overall survival from the time point at which primary hormonal therapy was commenced. When correlating response with other variables, complete and partial responders were grouped together with static disease as responders. It has previously been reported that patients with static disease on hormone therapy for 6 months have similar survival to patients with a partial response (Howell *et al*., 1988; Robertson *et al*., 1989).

### *Tissue samples*

Specimens consisted of either a primary tumour biopsy before treatment or a surgically resected primary carcinoma. Samples from all specimens were fixed in neutral buffered formalin for 24 h and processed routinely into paraffin blocks. Duplicate samples were immediately frozen in liquid nitrogen and maintained at −70°C. The various assays were performed at the National Hellenic Research Foundation, Athens (*ras* p21), Imperial Cancer Research Fund, London (p53) and Tenovus Institute for Cancer Research, Cardiff (ER, EGFR, Ki67, c-*erb*B-2). In most tumours sequential sections were used for measurement of these markers. When paraffin blocks were used for immunohistochemical staining, tissue sections 5 μm thick were mounted on slides and deparaffinised endogenous peroxidase activity was blocked by immersing the sections for 30 min in an aqueous solution of 3% hydrogen peroxide. All tumours were also analysed for histological type (Ellis *et al*., 1992), tumour grade (Elston and Ellis, 1991) and the presence of vascular invasion (Pinder *et al*., 1994).

### ras *p21*

The immunohistochemical analysis was performed with Y13259 which is a pan-ras antibody and recognises both normal and mutant p21, regardless of whether it is the product of H-*ras*, K-*ras* or N-*ras*. Immunostaining with Y13259 was carried out as previously reported (Papadimitriou *et al*., 1988). Briefly, deparaffinised sections were washed with phosphate-buffered saline (PBS) and treated with Y13-259 rat monoclonal antibody (diluted 1:100) for 90 min at 37°C. After washing with PBS they were treated with 1:100 biotinylated rabbit anti-rat IgG (Sigma) for 60 min at 37°C. Streptavidin-biotin conjugated peroxidase complex (Sigma, 1:100 in PBS) was applied for 30 min and peroxidase activity was visualised with diaminobenzene (Sigma). Sections were counterstained with haematoxylin and dehydrated. ras p21 was classified as either negative, low or intense staining. As in a previous report, for analysis of response, low and intense staining were combined as positive (Papadimitriou *et al*., 1988).

### *p53*

CMI, the polyclonal antibody against p53, was used to stain 3 μm paraffin sections on poly-L-lysine-coated slides. The CMI antibody is a rabbit high-titre polyclonal antiserum raised against human recombinant wild-type p53 protein. CMI antibody recognises both wild-type and mutant forms of the protein (Midgeley *et al*., 1992). Briefly we used a peroxidase conjugated streptavidin–biotin technique without antigen retrieved. The peroxidase reaction was demonstrated with diaminobenzene as chromogen with metal enhancement of the final colour reaction. Sections were counterstained with fast red (Barnes *et al*., 1993). p53 staining was assessed by two independent observers according to the percentage of cells staining and the overall intensity of the cells staining. Intensity was graded as negative (0) (no evidence of any positive staining in tumour cells), low (1) (weak staining in tumour cells only visible under the high power of the microscope), moderate (2) (positive staining of tumour cells evident under the low power of the microscope) and intense (3) (strong positive staining seen under the low power of the microscope). Proportional staining was classified as negative (0); <25% (1), 25–50% (2), 50–75% (3) and >75% (4). An overall value for p53 was then obtained as the product of intensity and percentage staining ranging from 0 to 12. Negative staining was taken as a p53 product of ⩽1 and all other values as positive for exploring the clinical correlations.

*C-erbB-2*

The PAb1 antibody to c-*erb*B-2 p185 (Triton Bioscience) was used according to the manufacturer's instructions. Staining was classified as positive if any of the tumour cell membranes stained with this antibody.

*EGFR*

EGFR-1, a mouse monoclonal anti-EGFR antibody (Amersham Ltd, Amersham, UK) was used to determine the expression of EGFR as previously reported (Nicholson *et al.*, 1994) on frozen section samples. In this publication we assessed EGFR as a predictor of endocrine response in breast cancer and established cut-off levels for the percentage of cells which stain positive. In the present report a negative result was considered when <20% of cells stained, mild when 20–60% stained and strongly positive when >60% cells stained. For analysis of clinical outcome, low and intense staining were combined as positive.

*Hormone receptor status*

The presence of ER was determined using Abbott ER-ICA monoclonal kit (Abbott Laboratories, North Chicago, USA), as previously described (Walker *et al.*, 1988; Robertson *et al.*, 1992). Briefly the ER-ICA employs rat anti-human ER antibody, H222. This antibody was incubated with a 5 μm frozen section of breast tumour and after washing was followed by a bridging antibody (goat anti-rat IgG) and finally a rat peroxidase–anti-peroxidase complex. Peroxidase activity was detected by the incubation of the antibody complex with diaminobenzene and hydrogen peroxide. Tumours were classified as ER negative by the ER-ICA if <5% of tumours cells stained positive.

The presence of PR was determined in the same manner as ER this time using Abbott PgR-ICA monoclonal kit (Abbott Laboratories). Tumours were classified as PR negative if <5% of tumour cells stained positive.

*Ki67 labelling index*

The antibody Ki67 recognises an antigen revealed in cell cycle. Frozen sections were air dried, fixed in acetone for 10 min and stained by the Ki67 monoclonal antibody (Dako Laboratories, Glostrup, Denmark) at a dilution of 1:15, followed by peroxidase-conjugated rabbit anti-mouse immunoglobulins at a dilution of 1:50. The peroxidase was demonstrated using hydrogen peroxide and diaminobenzene with imidazole enhancement to give a brown nuclear staining. Sections were counterstained with haematoxylin. Staining was recorded as percentage of positive tumour cell nuclei; 0–10% was considered negative, 11–29% mildly positive and 30% or more strongly positive. For analysis of clinical outcome, low and intense staining were combined as positive.

*Statistics*

Correlations between pathological and immunohistochemical variables and clinical response was examined using chi-square analysis or Fisher's exact test where appropriate. Plots of time to progression and overall survival were made using the method of Kaplan–Meier and univariate analysis of differences between groups carried out with the log-rank test. Multivariate analysis of variables affecting time to progression and overall survival were examined using a Cox regression model in a forward stepwise manner. A statistically significant difference was observed when $P<0.05$.

### Results

*Tumour biology*

In 41 (44.6%) of patients the index lesion was the primary breast tumour itself. The majority of patients had ductal carcinoma of no specific type (57.6%) and 20.6% had lobular carcinoma. Vascular invasion was definitely seen in 28.3% of patients. The majority of primary carcinomas were either grade 2 (42.4%) or grade 3 (54.3%).

Fifty-five patients (59.8%) were ER positive while PR positivity was seen in 41.3% of patients. A total of 85.9% of tumours were Ki67 positive and 60.9% were EGFR positive. The tumours of 66 patients (71.8%) stained positive for *ras* p21. Only 24 patients (26.1%) had tumours which stained positive for c-*erb*B-2. Overall, 39 patients (58%) had tumours which stained positive for p53.

*Response to hormonal therapy*

First-line hormonal therapy was tamoxifen in 64 patients (69.6%), Zoladex in six (6.5%), Zoladex plus tamoxifen in 19 (20.7%) and megesterol acetate in three (3.3%). Assessment by UICC criteria at 6 months showed complete response was seen in 11 (12%), partial in eight (8.7%) and static disease in

**Table I** Immunohistochemical parameters and clinical response

| *Variable* | *Category* | *Response (36)* | *Progression (56)* | P-*value* |
|---|---|---|---|---|
| Grade | 1 | 2 | 1 | |
| | 2 | 22 | 17 | |
| | 3 | 12 | 38 | 0.005 |
| Vascular invasion | No | 24 | 35 | |
| | Yes | 9 | 17 | |
| | Unknown | 3 | 4 | 0.85 |
| ER status | Negative | 3 | 34 | |
| | Positive | 33 | 22 | <0.001 |
| PR status | Negative | 15 | 38 | |
| | Positive | 21 | 17 | 0.02 |
| Ki67 status | Negative | 13 | 0 | |
| | Positive | 23 | 56 | <0.001 |
| EGFR status | Negative | 26 | 10 | |
| | Positive | 10 | 46 | <0.001 |
| c-*erb*B-2 status | Negative | 29 | 39 | |
| | Positive | 7 | 17 | 0.24 |
| p53 status | Negative | 15 | 24 | |
| | Positive | 21 | 32 | 0.91 |
| *ras* p21 status | Negative | 8 | 18 | |
| | Positive | 28 | 38 | 0.30 |

17 (18.5%). Within 6 months from initiation of therapy, 56 patients (61%) had progressed on hormonal therapy. The median time to progression following hormonal therapy was 5.0 months (range 1-86 months). At last follow-up, 80 patients (87%) had eventually progressed on hormonal therapy. Overall survival was a median of 25 months from commencing hormonal therapy with a range of 1-86 months. 76 patients (83%) had died at last follow-up.

*Relationship between immunohistochemical staining and clinical response*

Table I illustrates these relationships. There were 36 responders and 56 progressors at the 6 month assessment. The group of responders was significantly older and more likely to be post-menopausal than the progressors (mean age 60 *vs* 52; $P=0.002$). In addition, a clinical response was more likely to be observed in locally advanced tumours than in patients whose index lesion was a distant metastatic site. There were significantly more grade 3 carcinomas in the progressors ($P=0.005$), but lymphovascular invasion was not a predictor of progression.

As expected, ER negativity, PR negativity, a high Ki67 labelling index and positive EGFR staining all correlated with progression of disease on hormonal therapy. Total p53, *ras* p21 and c-*erb*B-2 staining did not correlate significantly with response to hormonal therapy. When the complete, partial and static disease was considered separately, there was no significant correlation with either the degree of *ras* p21 staining ($P=0.84$) or with total p53 expression ($P=0.52$).

**Figure 1** Kaplan-Meier plots for time to progression from commencement of hormonal treatment according to ER status, Ki67 index labelling, EGFR, c-*erb*B-2, p53 and *ras* p21 staining (O, positive; ■, negative). Univariate comparisons have been made with the log-rank test.

*Relationship between clinicopathological variables and* ras *p21 expression*

Of the 92 patients, 66 (71.8%) were positive for staining for the *ras* p21 product. There was no significant relationship between tumour grade, ER status, PR status, Ki67, EGFR or c-*erb*B-2. There was a significant relationship between both the intensity of p53 staining ($P = 0.006$) and the per cent staining ($P = 0.01$), but this significance showed only a trend when the p53 product was calculated and compared with *ras* p21 staining ($P = 0.06$).

*Relationship between clinicopathological variables and p53 expression*

Of the 92 patients, 53 (57.6%) were positive for p53. There was a trend for p53-positive tumours to be high grade ($P = 0.08$), have lymphovascular invasion ($P = 0.05$) and be ER positive ($P = 0.06$) and to stain *ras* p21 positive ($P = 0.06$). However, there was no relationship demonstrated for Ki67, EGFR or c-*erb*B-2.

*Relationship between clinicopathological variables and* c-erbB-2 expression

There was no correlation between c-*erb*B-2 and *ras* p21 status, p53 status or response to endocrine therapy.

*Time to progression and overall survival*

Figures 1 and 2 demonstrate the Kaplan–Meier plots for time to progression and overall survival for the different immunohistochemical parameters. On univariate analysis, ER

**Figure 2** Kaplan–Meier plots for overall survival from commencement of hormonal treatment according to ER status, Ki67 index labelling, EGFR, c-*erb*B-2, p53 and *ras* p21 staining (O, positive; ■, negative). Univariate comparisons have been made with the log-rank test.

negativity, Ki67 positivity and EGFR positivity were highly significant for both a shorter time to progression and a poor overall survival ($P<0.001$). However, the staining of the original primary tumour for c-*erb*B-2, p53 and *ras* p21 did not correlate significantly with time to progression and overall survival.

All relevant factors were examined for their influence on time to progression and overall survival using the Cox regression model. ER status ($P<0.001$), Ki67 ($P=0.006$) and EGFR ($P=0.004$) were the only factors found to be statistically significant independent predictors of time to progression. For overall survival, only ER status ($P=0.008$) and Ki67 labelling index ($P=0.003$) were independent covariates.

### Discussion

The aim of the study was an attempt to identify one or more oncoproteins whose expression may serve as a guide to therapy as well as a prognostic indicator in advanced breast cancer. Examination of *ras* p21, p53 and c-*erb*B-2 showed no correlation with either clinical response, time to progression or overall survival from the date on which hormone treatment was started.

The validity of the series is confirmed by the observation that the clinical outcome variables were highly related to ER status, Ki67 and EGF receptor status. In primary breast carcinomas ER status is a measure of endocrine responsiveness (Hawkins *et al*., 1987; Low *et al*., 1992) while Ki67 and EGFRs are related to the proliferative rate of the primary tumour (Sainsbury *et al*., 1987; Locker *et al*., 1992). Our data imply that the tumour retains its same biological characteristics in the locally advanced and metastatic phases.

The finding that there was no significant correlation between *ras* p21 expression and either tumour histology or the other immunohistochemical parameters is of interest in light of previous research. Work by Ohuchi *et al*. (1986) and Going *et al*. (1992) indicates that *ras* p21 expression increases through the histological progression from normal breast epithelium to *in situ* cancer. There is little further increase in invasive cancer and metastases have a rather heterogeneous staining, implying that the expression of *ras* p21 is not required for maintenance of the transformed phenotype (Fromowitz *et al*., 1987). There was early evidence that enhanced *ras* p21 expression was associated with rapidly proliferating, high-grade tumours with lymph node metastases, but others have failed to show any relationship with histological type, tumour grade, hormone receptor status, tumour diameter, lymph node status or vascular invasion (Lundy *et al*., 1986; Spandidos *et al*., 1989). In conjunction with the present study in advanced breast cancer, there is little support for that premise that *ras* p21 expression is associated with aggressive tumour behaviour.

In a previous study by our group of stage I and II breast cancers expression of stabilised p53 protein correlated significantly with markers of poor prognosis – i.e. high tumour grade, expression of EGFR and c-*erb*B-2 protein overexpression (Poller *et al*., 1992). In the present series, there was a trend to such an association with grade and vascular invasion, but not with either the EGFR or c-*erb*B-2. In our previous study (Poller *et al*., 1992) p53 expression showed only a weak link with patient survival. Other studies have reported a stronger correlation between p53 expression and poor prognosis (Thor *et al*., 1992; Allred *et al*., 1993; Barnes *et al*., 1993; Goldschmid *et al*., 1994) and even that it is independently significant on multivariate analysis (Thor *et al*., 1992).

In our previous study of stage I and II disease (Poller *et al*., 1992) we reported a strong inverse relationship of p53 expression with ER status. In the present study there was a non-significant trend for ER-positive tumours to be p53 positive. One possible explanation for this difference between our two studies may be that the present study involves a smaller, more select group of patients who have identified their poorer prognosis by presenting with a locally advanced primary cancer or by developing symptomatic metastases. In these particular tumours ER positivity is not associated with as good a prognosis as ER positivity in stage I and II tumours, some of which will never recur. It may not be too surprising therefore that the tumours in the present study express p53 and ER together.

It is difficult to interpret the trend for p53-positive tumours to be *ras* p21 positive as well, in view of the fact that *ras* p21 does not correlate with any of the other parameters associated with rapid cellular proliferation. One explanation may be that in previous series there was a much larger proportion of patients with grade 1 tumours, while in this study the vast majority were grade 2 or 3. This clustering of grades may have reduced the magnitude of the differences previously observed.

C-*erb*B-2 did not correlate with either *ras* p21 or p53 oncoprotein expression. In this same series of patients we have previously reported that c-*erb*B-2 does not correlate with EGFR or Ki67 (Nicholson *et al*., 1993). *In vitro* studies have shown that in ER-positive breast cancer cell lines the expression of c-*erb*B-2 is oestradiol regulated. Oestradiol stimulated cell proliferation while at the same time it down-regulated the expression of c-*erb*B-2 in ER-positive cell lines MCF-7 and T47D (Dati *et al*., 1990) and MCF-7 and ZR-75.1 (Russell and Hung, 1992). Further studies by the first of these two groups reported that the anti-oestrogen tamoxifen inhibited cell growth and enhanced c-*erb*B-2 expression in ER-positive cell lines T47D and ZR75.1. Tamoxifen had no effect on cell growth or c-*erb*B-2 expression in the ER-negative cell line MDA.MB231 (Antoniotti *et al*., 1992). The other group has shown that while oestradiol had no effect on the growth of c-*erb*B-2 expression of the ER-negative cell line BT-474, the addition of ER to the cell line BT-474 was sufficient to allow oestradiol to repress the expression of c-*erb*B-2 (Russell and Hung, 1992). It is therefore interesting that in the present study expression of c-*erb*B-2 by the primary tumour did not correlate with response to endocrine therapy.

c-*erb*B-2 expression did not correlate with patient survival. This is contrary to a previous publication looking at a similar number but different group of patients with advanced breast cancer in which we reported that c-*erb*B-2 expression did correlate with survival (Lovekin *et al*., 1991). It is unclear from the apparently conflicting results of our two studies whether c-*erb*B-2 expression does correlate with survival in patients with advanced breast cancer.

In conclusion, immunostaining of the primary tumour with monoclonal antibodies to *ras* p21, c-*erb*B-2 and p53 either separately or as a panel of stains did not in this study provide a useful predictor of response to hormonal therapy in locally advanced or metastatic breast cancer. Furthermore, these parameters bore no relationship to either the time to progression or the overall survival from the time of instituting hormonal therapy. These findings, particularly for c-*erb*B-2, do not appear to be in keeping with *in vitro* data suggesting c-*erb*B-2 expression may be hormonally regulated, at least in ER-positive breast cancer cell lines. It would be interesting to note the effect of tamoxifen on c-*erb*B-2 expression of tumours by sequential biopsies. This is currently the subject of ongoing studies.

**Acknowledgement**
We are grateful to Tenovus Institute, Cardiff, for their financial support, which funded some of the laboratory data.

### References

ALI IV, CAMPBELL G, LIDERAU R AND CALLAHAN R. (1988). Lack of evidence for the prognostic significance of c-erbB-2 amplification in human breast carcinoma. *Oncogene Res.*, **3**, 139-144.

ALLRED DC, CLARK GM, ELLEDGE R, FUQUA SAW, BROWN RW, CHAMNESS GC, OSBORNE CK AND MCGUIRE WL. (1993). Association of p53 protein expression with tumour cell proliferation rate and clinical outcome in node-negative breast cancer. *J. Natl Cancer Inst.*, **85**, 200-206.

ANBAZHAGAN R, GELBER RD, BETTELHEIM R, GOLDHIRSCH A AND GUSTERSON BA. (1991). Association of c-erbB-2 expression and S-phase fraction in the prognosis of node positive breast cancer. *Annals of Oncology*, **2**, 47-53.

ANTONIOTTI S, MAGGIORA P, DATI C AND DEBORTOLI M. (1992). Tamoxifen up-regulates breast cancer cells in vitro. *Eur. J. Cancer*, **28**, 318-321.

BARNES DM, DUBLIN EA, FISHER J, LEVISON DA AND MILLIS RR. (1993). Immunohistochemical detection of p53 protein in mammary carcinoma: an important new independent indicator of prognosis? *Hum. Pathol.*, **24**, 469-476.

BARTEK J, BARTKOVA J, VOJTESEK B, STASKOVA Z, REJTHAR A, KOVARIK J AND LANE DP. (1990). Patterns of expression of the p53 tumour suppressor in human breast tissues and tumours in situ and in vitro. *Int. J. Cancer*, **46**, 839-844.

BENNETT WP, HOLLSTEIN MC, METCALF RA, WELSH JA, HE A, ZHU S, KUSTERS I, RESAU JH, TRUMP BF, LANE DP AND HARRIS CC. (1992). Mutation and protein accumulation during multistage human oesophageal carcinogenesis. *Cancer Res.*, **52**, 6092-6097.

BRITISH BREAST GROUP. (1974). Assessment of response to treatment in advanced breast cancer. *Lancet*, **2**, 38-39.

DATI C, ANTONIOTTI S, TAVERNA D, PERROTEAU I AND DE BORTOLI M. (1990). Inhibition of c-erbB2 oncogene expression by estrogens in human breast cancer cells. *Oncogene*, **5**, 1001-1006.

ELLIS IO, GALEA MH, BROUGHTON N, LOCKER A, BLAMEY RW AND ELSTON CW. (1992). Pathological prognostic factors in breast cancer. II. Histological type. Relationship with survival in a large study with long term follow up. *Histopathology*, **20**, 479-489.

ELSTON CW AND ELLIS IO. (1991). Pathological prognostic factors in breast cancer. I. The value of histological grade in breast cancer; experience from a large study with long-term follow-up. *Histopathology*, **19**, 403-410.

ERNBERG IT. (1990). Oncogenes and tumor growth factors in breast cancer. *Acta Oncol.*, **29**, 331-334.

FISHER CJ, GILLETT CE, VOTESEK B, BARNES DM AND MILLIS RR. (1994). Problems with p53 immunochemical staining: the effect of fixation and variation in the methods of evaluation. *Br. J. Cancer*, **69**, 26-31.

FROMOWITZ FB, VIOLA MV, CHAO S, ORAVEZ S, MISHRIKI Y, FINKEL G, GRIMSON R AND LUNDY J. (1987). ras p21 expression in the progression of breast cancer. *Hum. Pathol.*, **18**, 1268-1275.

GOING JJ, ANDERSON TJ AND WYLLIE AH. (1992). Ras p21 in breast tissue: associations with pathology and cellular localisation. *Br. J. Cancer*, **65**, 45-50.

GOLDSCHMIDT RA, MERKEL DE, VILLA D, WINCHESTER DJ, FENDELMAN J, GATBUTTON C AND RADEMAKER AW. (1994). Overaccumulation of p53 protein and tumour diameter as predictors of recurrence for patients with node-negative infiltrating ductal carcinoma of the breast. *Proc. ASCO*, **13**, 73.

GULLICK WJ, LOVE SB, WRIGHT C, BARNES DM, GUSTERSON B, HARRIS AL AND ALTMAN DG. (1991). C-erbB2 protein overexpression in breast cancer is a risk factor in patients with involved and uninvolved lymph nodes. *Br. J. Cancer*, **63**, 434-438.

GUSTERSON BA, ANBAZHAGAN K, WARREN W *et al.* (1991). Expression of p53 in premalignant and malignant squamous epithelium. *Oncogene*, **6**, 1785-1789.

GUSTERSON BA, GELBER RD, GOLDHIRSCH A, PRICE KN, SODERBORGH JS, ANBAZHAGAN R, STYLES J, RUDENSTORM CM, GOLOUH R, REED R, MARTINEZ-TELLO F, TILTMAN A, TORHORST J, GRIGOLATO P, BETTELHEIM R AND NEVILLE AM. (1992). Prognostic importance of c-erbB2 expression in breast cancer. *J. Clin. Oncol.*, **10**, 1049-1056.

HALL JM, ZUPPAN PJ, ANDERSON LA, HUEY B, CARTER C AND KING MC. (1989). Oncogenes and human breast cancer. *Am. J. Hum. Genet.*, **44**, 577-584.

HALL PA, MCKEE PH, MENAGE H, DU P, DOVER R AND LANE DP. (1993). High levels of p53 protein in UV-irradiated normal human skin. *Oncogene*, **8**, 203-207.

HAWKINS RA, TESADALE AL, FERGUSON WA AND GOING JJ. (1987). Oestrogen receptor activity in intraduct and invasive breast carcinomas. *Breast Cancer Res. Treat.*, **9**, 129-133.

HAYWARD JL, CARBONE PP, HEUSON JC, KUMAOKA S, SEGALOFF A AND RUBENS RD. (1977). Assessment of response to therapy in advanced breast cancer: A project of the programme on clinical oncology of the international union against cancer, Geneva, Switzerland. *Cancer*, **39**, 1289-1294.

HOLLSTEIN M, SIDRANSKY D, VOGELSTEIN B AND HARRIS CC. (1991). p53 mutations in human cancer. *Science*, **253**, 49-53.

HORAK L, SMITH K, BROMLEY L, LEJEUNE S, GREENALL M, LANE D AND HARRIS AL. (1991). Mutant p53, EGF receptor and c-erbB-2 expression in human breast cancer. *Oncogene*, **6**, 2277-2284.

HOWELL A, MACKINTOSH J, JONES M, REDFORD J, WAGSTAFF J AND SELLWOOD RA. (1988). The definition of the 'no change' category in patients treated with endocrine therapy and chemotherapy for advanced carcinoma of the breast. *Eur. J. Cancer Clin. Oncol.*, **24**, 1567-1572.

LOCKER AP, BIRRELL K, BELL JA, NICHOLSON RI, ELSTON CW, BLAMEY RW AND ELLIS IO. (1992). Ki67 immunoreactivity in breast carcinoma: relationships to prognostic variables and short term survival. *Eur. J. Surg. Oncol.*, **18**, 224-229.

LOVEKIN C, ELLIS IO, LOCKER A, ROBERTSON JFR, BELL J, NICHOLSON R, GULLICK WJ, ELSTON CW AND BLAMEY RW. (1991). c-erbB-2 oncoprotein expression in primary and advanced breast cancer. *Br. J. Cancer*, **63**, 439-443.

LOW SC, DIXON AR, BELL J, ELLIS IO, ELSTON CW, ROBERTSON JFR AND BLAMEY RW. (1992). Tumour oestrogen receptor content allows selection of elderly patients with breast cancer for conservative tamoxifen treatment. *Br. J. Surg.*, **79**, 1314-1316.

LUNDY J, GRIMSON R, MISHRIKI Y, CHAO S, ORAVEZ S, FROMOWIZT F AND VIOLA NV. (1986). Elevated ras oncogene expression correlates with lymph node metastases in breast cancer patients. *J. Clin. Oncol.*, **14**, 1321.

MANGUES R, KAHN JM, SEIDMAN I AND PELLICER A. (1994). An overexpressed N-ras proto-oncogene co-operates with N-methylnitrosourea in mouse mammary carcinogenesis. *Cancer Res.*, **54**, 6395-6401.

MIDGELEY CA, FISHER C, BARTEK J, VOJTESEK B, LANE DP AND BARNES DM. (1992). Analysis of p53 expression in human tumours: an antibody raised against human p53 expression in escherishia coli. *J. Cell Sci.*, **101**, 183-189.

NICHOLSON RI, BOUZUBAR N, WALKER KJ, MCCLELLAND R, DIXON AR, ROBERTSON JFR, ELLIS IO AND BLAMEY RW. (1991). Hormone sensitivity in breast cancer: influence of heterogeneity of oestrogen receptor expression and cell proliferation. *Eur. J. Cancer*, **27**, 908-913.

NICHOLSON RI, MCCLELLAND RA, FINLAY P, EATON CL, GULLICK WJ, DIXON AR, ROBERTSON JFR, ELLIS IO AND BLAMEY RW. (1993). Relationship between EGF-R, c-erbB2 protein expression and Ki67 immuno-staining in breast cancer and hormone sensitivity. *Eur. J. Cancer*, **29A**, 1018-1023.

NICHOLSON RI, MCCLELLAND RA, GEE JMW, MANNING DL, CANNON P, ROBERTSON JFR, ELLIS IO AND BLAMEY RW. (1994). Epidermal growth factor receptor expression in breast cancer: Association with response to endocrine therapy. *Br. Cancer Res. Treat.*, **29**, 117-125.

OHUCHI N, THOR A, PAGE DL, HAND PH, HALTER SA AND SCHLOM J. (1986). Expression of the 21,000 molecular weight ras protein in a spectrum of benign and malignant human mammary tissues. *Cancer Res.*, **46**, 2511-2519.

O'REILLY SM, BARNES DM, CAMPLEJOHN RS, BARTKOVA J, GREGORY WM AND RICHARDS MA. (1991). The relationship between c-erbB2 expression, S-phase fraction and prognosis in breast cancer. *Br. J. Cancer*, **63**, 444-446.

PAPADIMITRIOU K, YIAGNISIS M, TOLIS G AND SPANDIDOS DA. (1988). Immunohistochemical analysis of the ras oncogene product in human thyroid neoplasms. *Anticancer Res.*, **8**, 1223-1228.

PIGNATELLI M, STAMP GWH, KAFIRI G, LANE D AND BODMER WF. (1992). Overexpression of p53 nuclear oncoprotein in colorectal adenomas. *Int. J. Cancer*, **50**, 683-688.

PINDER SE, ELLIS IO, GALEA M, O'ROURKE W, BLAMEY RW AND ELSTON CW. (1994). Pathological prognostic factors in breast cancer. III. Vascular invasion: Relationship with recurrence and survival in a large study with long term follow-up. *Histopathology*, **24**, 41-47.

POLLER DN, HUTCHINGS CE, GALEA M, BELL JA, NICHOLSON RI, ELSTON CW, BLAMEY RW AND ELLIS IO. (1992). p53 protein expression in human breast carcinoma: relationship of expression to epidermal growth factor receptor, c-erbB-2 protein overexpression, and oestrogen receptor. *Br. J. Cancer*, **66**, 583-588.

RASBRIDGE SA, GILLETT CE, SEYMOUR AM AND MILLIS RR. (1993). The effect of chemotherapy on histological and biological features of breast cancer. *J. Pathol.*, **169** (suppl.), 191.

ROBERTSON JFR, WILLIAMS MR, TODD J, NICHOLSON RI, MORGAN DAL AND BLAMEY RW. (1989). Factors predicting the response of patients with advanced breast cancer to endocrine (Megace) therapy. *Eur. J. Cancer Clin. Oncol.*, **25**, 469-475.

ROBERTSON JFR, BATES K, PEARSON D, BLAMEY RW AND NICHOLSON RI. (1992). Comparison of two oestrogen receptor assays in the prediction of the clinical course of patients with advanced breast cancer. *Br. J. Cancer*, **65**, 727-730.

RUSSELL KS AND HUNG HC. (1992). Transcriptional repression of the new proto-oncogene by estrogen stimulated estrogen receptor. *Cancer Res.*, **52**, 6624-6629.

SAINSBURY RJ, FARNDON JR, NEEDHAM GK, MALCOLM AJ AND HARRIS AL. (1987). Epidermal growth factor receptor status as a predictor of early recurrence and death from breast cancer. *Lancet*, **1**, 1398-1402.

SLAMON DJ, CLARK GM, WONG SG, LEVIN WJ, ULLRICH A AND McGUIRE WKL. (1987). Human breast cancer: correlation of relapse and survival with amplification of the HER-2/neu oncogene. *Science*, **235**, 177-182.

SPANDIDOS A AND AGNANTIS NJ. (1984). Human malignant tumours of the breast, as compared to their respective normal tissue, have elevated expression of the Harvey ras oncogene. *Anticancer Res.*, **4**, 269-272.

SPANDIDOS DA, YIAGNISIS M, PAPADIMITRIOU K AND FIELD JK. (1989). Ras, c-myc and c-erb-2 oncoproteins in human breast cancer. *Anticancer Res.*, **9**, 1385-1394.

THOR AM, MOORE DH, EDGERTON SM, KAWASAKI ES, REIHSAUS E, LYNCH HT, MARCUS JN, SCHWARTZ L, CHEN LC, MAYALL BH AND SMITH HS. (1992). Accumulation of p53 tumour suppressor gene protein: an independent marker of prognosis in breast cancers. *J. Natl Cancer Inst.*, **84**, 845-855.

THOR A, OHUCHI N, HAND PH, CALLAHAN R, WEEKS MO, THEILLET C, LIDEREAU R, ESCOT C, PAGE DL, VILASI V AND SCHLOM J. (1986). ras gene alterations and enhanced levels of ras p21 expression in a spectrum of benign and malignant human mammary tissues. *Lab. Invest.*, **55**, 603-615.

WALKER KJ, BOUZABAR N, ROBERTSON JFR, ELLIS IO, ELSTON CW, BLAMEY RW, WILSON DW, GRIFFITHS K AND NICHOLSON RI. (1988). Immunocytochemical localisation of estrogen receptors in human breast tissue. *Cancer Res.*, **48**, 6517-6522.

WATSON DMA, ELTON RA, HACK WJL, DIXON JM, CHETTY U AND MILLER WR. (1990). The H-ras oncogene product p21 and prognosis in human breast cancer. *Br. Cancer Res. Treat.*, **17**, 161-169.

WINSTANLEY J, COOKE T, MURRAY GD, PLATT-HIGGINS A, GEORGE WD, HOLT S, MYSKOV M, SPEDDING A, BARRACLOUGH BR AND RUDLAND PS. (1991). The long-term prognostic significance of c-erbB2 in primary breast cancer. *Br. J. Cancer*, **63**, 447-450.

WRIGHT C, ANGUS B, NICHOLSON S, RICHARD J, SAINSBURY C, CAIRNS J, GULLICK WJ, KELLY P, HARRIS AL AND WILSON HORNE CH. (1989). Expression of c-erbB-2 oncoprotein: A prognostic indicator in human breast cancer. *Cancer Res.*, **49**, 2087-2090.

ZHOU DJ, AHUJA H AND CLINE MT. (1989). Proto-oncogene abnormalities in human breast cancer. c-erbB-2 amplification does not correlate with recurrence of disease. *Oncogene*, **4**, 105-108.